# Risk factors of sudden cardiac death in Egyptian patients younger than 40 years

**DOI:** 10.1186/s43044-023-00373-2

**Published:** 2023-06-07

**Authors:** Ahmed Nabil Ali, Hend Ali Abdeltawab, Hayam Eldamanhoury, Mervat Aboulmaaty

**Affiliations:** Cardiology Department, Faculty of Medicine, Ain Shams University, Cairo, Egypt

**Keywords:** Dilated cardiomyopathy, Hypertrophic cardiomyopathy, Long QT, Positive family history, Young age

## Abstract

**Background:**

Sudden cardiac death in young people is a major problem. The causes are well known; however, they may not be discovered before the episode of sudden death. A challenge for the future is identifying patients at risk before an episode of sudden cardiac death. Development of preventive and educational programs is required to identify sudden cardiac death/sudden cardiac arrest (SCD/SCA) risk factors, causes and characteristics. We aimed to study the characteristics of SCD/SCA in a cohort of young Egyptian population. Our retrospective cohort study included 246 patients of SCD/SCA who were collected from 5000 records of arrhythmia patients from January 2010 till January 2020. The records of the specialized arrhythmia clinic were reviewed to collect the families of SCD/SCA. All patients and/or their first-degree relatives were subjected to thorough history taking and clinical evaluation and investigations. Comparisons were done regarding age group and presence of positive family history of SCD.

**Results:**

Males constituted 56.9% of the study population. Mean age was 26.6 ± 12.73 years. Positive family history was present in 202 (82.1%) cases. Sixty-one percent of the cases had history of syncopal attacks. SCD/SCA during non-exertion or sleep occurred in 50.4% of cases. Hypertrophic cardiomyopathy was the most common cause of SCD/SCA (20.3%), followed by dilated cardiomyopathy (19.1%), long QT Syndrome (11.4%), complete heart block (8.5%), and Brugada syndrome (6.8%). In the older age group of 18–40 years, hypertrophic cardiomyopathy was responsible for SCD in 44 patients (25.3%) versus 6 patients (8.3%) in younger age group (*p*-value: 0.003). DCM was also dominant in older age group (42 patients; 24.1%) versus 5 patients (6.9%) in younger age group. Hypertrophic cardiomyopathy was more prevalent in positive family history group (46 patients; 22.8%) versus 4 patients (9.1%) in negative family history group (*p*-value: 0.041).

**Conclusion:**

Family history of SCD was the most common risk factor of SCD. The most common cause of SCD in young Egyptian patients below 40 years was hypertrophic cardiomyopathy, followed by dilated cardiomyopathy. Both diseases were more common in the age group between 18 and 40 years. Hypertrophic cardiomyopathy was more common in patients with positive family history of SCD/SCA.

## Background

Sudden cardiac death (SCD) is an important problem with multiple etiologies and risk factors. Most studies refer to SCD as a sudden death from a cardiovascular cause which occurs within 1 h of the onset symptom when witnessed, or if not witnessed, an unexpected death from a cardiovascular cause where the individual was observed to be alive within the previous 24 h [1].

However, sudden cardiac arrest (SCA), defined as a natural and unexpected collapse of presumed cardiac etiology, is a major contributor to cardiovascular mortality [1]. Sudden cardiac death remains a major cause of mortality, accounting for 10–20% of deaths. The annual incidence of SCD is estimated as 1–1.8 in 100,000 in a subject, approximately more than 3.7 million per year around the world [2]. Potential number of SCD is caused by cardiomyopathies, primary electrical diseases, conduction system disorders, inherited heart diseases and abnormal heart rhythms [3]. Primary electrical diseases are group of inherited diseases that can lead to various cardiac arrhythmias which can cause sudden cardiac death. These diseases are caused by inherited genetic abnormalities that alter the cell ionic and electrical behavior. Primary electrical diseases include long QT syndrome, short QT syndrome, Brugada syndrome, and catecholaminergic polymorphic ventricular tachycardia [3].

The presence of prior cardiac disease increases the annual incidence of SCD, being 1/1000 per year in the general population, and increasing to 5% per year in the patient with a previous coronary event, up to 15% in the patient with impaired ejection fraction less than 35%, and finally 20% in any survivor from cardiac arrest [4]. There is a great challenge in diagnosing cases at risk for SCD because it usually occurs in candidates with previously normal medical examination [4].

In many cases, SCD can be the first presentation of an underlying cardiac condition. So, it is important to know families who have a history of sudden cardiac death and try to identify risk factors to reduce the burden of SCD [4].

Cardiac assessment of family members of patients who experienced SCD is very important as many causes of SCD have familial relationship. Screening of these families are strongly encouraged with special emphasis on features that suggest a predisposing disorder that could cause SCD, including coronary artery disease, cardiomyopathy, or any inherited primary electrical disorder [4, 5].

Our aim was to study causes of death in a cohort of Egyptian population who died suddenly or were resuscitated from SCA and attend to follow up at arrhythmia clinic at our institution.

## Methods

The present work is a retrospective study including 246 patients who died or were resuscitated from sudden cardiac death. The data of these patients and their families were collected from 5000 records of arrhythmia patients followed up at a specialized arrhythmia clinic in the period from January 2010 till January 2020.

The study included patients with ages from 2 to 40 years, death situation fulfilling the definition of SCD as mentioned before [1]. While death cases due to any accidents and patients below 2 years or above 40 years were excluded from the study.

The following data were collected from revising the medical records: personal history: (name, age, sex, body mass index (BMI), lifestyle, occupation, marital status, socioeconomic status, risk factors as: smoking, addiction to drugs that can increase the risk for SCD as: cocaine, marijuana, heroin, and opioids). Socioeconomic status was classified into three levels (high, moderate, and low). High socioeconomic status was determined when the average income per year was more than 120,000 Egyptian pounds, moderate socioeconomic status when the average income per year ranged between 60,000 and 120,000 Egyptian pounds, and low socioeconomic status when the average income per year was less than 60,000 Egyptian pounds.

Medical history included circumstances of death: (pre-syncope, syncope, exercise, palpitation, breathlessness, sleep, rest, any other triggers), family history of sudden cardiac death, history of unexplained syncope, any arrhythmias, devices implantation, electrophysiological study and ablation, and coronary artery disease.

Clinical data of the SCD victims and survivors of their families were collected from medical files. Available investigations, including ECG, echocardiography, the results of previous stress tests done for any documented CPVT or PVCs or VTs or bradyarrhythmias by exercise, were reviewed. Coronary angiography or multi-slice CT coronary angiography was done for survivors of SCD in suspected cases to exclude ischemia or coronary anomalies. Cardiac MRI (in survivors of SCD) was reviewed to document or exclude cases of ARVD, HCM. Holter recordings and laboratory investigations previously done (CBC, thyroid function test, potassium level, magnesium level, serum creatinine, BUN) were reviewed.

### Ethical considerations

This study was approved by our institution’s ethical committee according to the ethical guidelines of the 1975 Declaration of Helsinki as revised in 2008. The data of the families and victims were secured and were hidden during publishing of study results.

### Statistical methods

Analysis of data was performed using Statistical Package for Social Science (IBM SPSS version 23). Description of variables was presented as follows: Description of quantitative variables was in the form of mean, standard deviation (SD), minimum and maximum. Description of qualitative variables was in the form of numbers (No.) and percent’s (%). Data were explored for normality using Kolmogorov–Smirnov test of normality. The results of Kolmogorov–Smirnov test indicated that most of data were normally distributed (parametric data), so parametric tests were used for most of the comparisons. Comparison between quantitative variables was carried out by One-way analysis of variance (ANOVA) which was used to test the difference between the means of several subgroups of a variable. Comparison between qualitative variables was carried out by Chi-square test, which was used to test the statistical significance of differences in a classification system (one-way classification) or the relationship between two classification systems (two-way classification). The significance of the results was assessed and considered significant when *p*-value ≤ 0.05.

## Results

Our retrospective cohort study included 246 patients of SCD/SCA whose records were collected and reviewed from 5000 records of arrhythmia patients followed up at a specialized arrhythmia clinic in the period from January 2010 till January 2020. The study included patients with an outcome of survival from SCD (survivors) who sought medical advice in specialized arrhythmia clinic and received the proper treatment according to the etiology of SCD (including ICD implantation & invasive electrophysiological studies with ablation when needed). It also included patients with an outcome of cardiac death who were either followed up and diagnosed before their death or were retrogradely diagnosed by revising their investigations in arrhythmia clinic during assessment of their relatives and family members who had similar cardiac conditions predisposing to SCD. None of the patients with death outcome had an autopsy done. The mean age was 26.6 ± 12.73 years. Demographic data of the studied group are detailed in Table 1. Sixty-nine patients (28.8%) were receiving beta-blockers, 24 patients (9.7%) were receiving amiodarone, while 95 patients (38.6%) were receiving anti-failure medications. It was noted that an event of SCD in at least one family member was present in 202 cases (82.1% of the study population).

**Table 1:** Demographic data of our studied population:

Demographic data		Total (*n *= 246)
Age (years)	Mean ± SD	26.5 ± 12.73
	Range	2–40
Gender	Males	140 (56.9%)
	Females	106 (43.1%)
BMI (kg/m^2^)	Mean ± SD	24.17 ± 1.8
	Range	19.5–26.7
Socioeconomic status		
High	No. (%)	31 (12.6%)
Moderate		170 (69.1%)
Low		45 (18.3%)
Residency		
Urban areas	No. (%)	138 (56%)
Rural areas		108 (43.5%)
FH OF CAD		
HTN	HTN	20 (8%)
DM	DM	8 (3.2%)
Smoker	Smoker	10 (4%)
Addiction	Addiction	1 (0.4%)
Consanguinity	No. (%)	100 (40.7%)
Positive family history of SCD	No. (%)	202 (82.1%)

*BMI* Body mass index, *CAD* Coronary artery disease, *DM* Diabetes mellitus, *FH* Family history, *HTN* Hypertension, *SCD* Sudden cardiac death

### Circumstances of the event

Repeated syncopal attacks occurred in 151 cases (61.3%). Thirty-eight patients (15.4%) developed SCD/SCA during practicing exercise (36 patients during practicing sports and 2 patients during exercise ECG). Two patients developed SCD during sleep and 84 patients developed SCD/SCA during regular activity, while 122 patients (49.6%) developed SCD/SCA at home without any exercise.

### Etiology of SCD

**Fig. 1:**
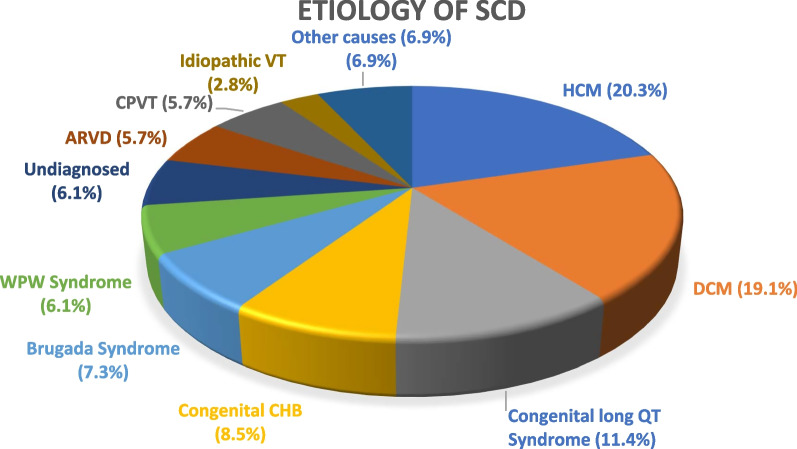
Showing different etiologies of SCD in our studied population. *ARVD* Arrhythmogenic right ventricular dysplasia, *CHB* Complete heart block, *CPVT* catecholamine-mediated ventricular tachycardia, *DCM* Dilated cardiomyopathy, *HCM* Hypertrophic cardiomyopathy, *VT* Ventricular tachycardia, *WPW* Wolff–Parkinson–White

Hypertrophic cardiomyopathy (HCM) was the most common etiology of SCD in our study presented by 50 patients (20.3%), followed by dilated cardiomyopathy (DCM) in 47 patients (19.1%), and congenital long QT Syndrome in 28 patients (11.4%) (Fig. 1). Less common etiologies included congenital CHB in 21 patients (8.5%), Brugada syndrome in 18 patients (6.8%), WPW syndrome in 15 patients (6.1%). Other causes included primary VF in 5 patients, concealed anteroseptal accessory pathway in 3 patients, PVCs arising from LVOT in 3 patients, ischemic heart disease in 2 patients, mitral annular disjunction in 2 patients, and LV non-compaction in only 2 patients. 6.1% of patients were undiagnosed.

Ninety patients (36.5%) received implantable devices in the form of 58 ICD devices, 20 CRT-D devices, 4 CRT-P devices, and 8 permanent pacemakers in the form of DDD devices. All defibrillator devices were implanted as a secondary prevention for SCD survivors. Diagnoses of patients and indications of implantation are listed in Table 2. Invasive electrophysiological study was done in 18 patients after the event of SCD. They were done aiming to reach the diagnosis and/or properly treat the cause by radiofrequency ablation. One patient underwent ventricular tachycardia (VT) ablation, while VT was not induced in other 3 patients. Eleven patients with WPW syndrome underwent successful ablation of accessory pathway (2 patients of them had right antero-septal accessory pathway, 3 patients had postero-septal accessory pathway, while 4 patients had left lateral accessory pathway, in addition to 2 patients who had successful ablation of concealed antero-septal accessory pathway). One patient had parahisian accessory pathway which was not ablated. RVOT PVCs was ablated in one patient, while LVOT PVCs was ablated in another patient.

**Table 2:** Device implantation in our studied population

	Total number	ICD	CRT-D	CRT-P	DDD
Total (*n* = 90)	90 (36.5%)	58 (23.5%)	20 (8.1%)	4 (1.6%)	8 (3.2%)
HCM	20	11	9		
DCM	34	21	9	4	
LV non compaction	2	1	1		
Mitral annular disjunction	1	1			
Primary VF	4	4			
Idiopathic VT	3	3			
ARVD	6	6			
Long QT Syndrome	5	5			
Brugada Syndrome	5	5			
ICM	1	1			
CHB	9		1		8

*ARVD* Arrhythmogenic right ventricular dysplasia, *CHB* Complete heart block, *CRT-D/P* Cardiac resynchronization therapy defibrillator/pacemaker, *DCM* Dilated cardiomyopathy, *DDD* Dual chamber pacemaker, *HCM* Hypertrophic cardiomyopathy, *ICD* Implantable cardioverter defibrillator, *ICM* Ischemic cardiomyopathy, *LV* Left ventricle, *VF* Ventricular fibrillation, *VT* Ventricular tachycardia

### Comparison according to age

Study population was divided into 2 groups according to age into younger age group (from 2 to 18 years) which included 72 patients (29.3%) and older age group (from 18 to 40 years) which included 147 patients (70.7%). Regarding demographic data, both groups were similar in terms of gender with no significant difference between both groups. Mean BMI was significantly lower in younger age group (p value: 0.041). Positive parents’ consanguinity was found in 41 patients (56.9%) in younger age group which was significantly lower than older age group (33.9.7%) with a *p*-value of 0.001 (Table 3).

**Table 3:** Comparing demographic data between younger and older age groups

Demographic data		Younger age group (*n* = 72)	Older age group (*n* = 147)	*p*-value
Gender	Males No. (%)	34 (47.2%)	106 (69.9%)	0.0489
	Females No. (%)	38 (52.8%)	68 (39.1%)	
BMI (kg/m^2^)	Mean ± SD	20.15 ± 1.7	22.5 ± 1.31	0.041*
	Range	19.5–24.6	20.8–26.7	
Socioeconomic status	High No. (%)	20 (27.8%)	11 (6.3%)	0.0012*
	Moderate No. (%)	30 (41.7%)	140 (80.4%)	
	Low No. (%)	22 (30.5%)	23 (13.2%)	
Residency	Urban areas No. (%)	30 (41.7%)	108(62.1%)	< 0.0001*
	Rural areas No. (%)	42 (58.3%)	66 (37.9%)	
Consanguinity	No. (%)	41 (56.9%)	59 (33.9%)	0.0008*

*BMI* Body mass index

*P < 0.05 is considered significant

There was no difference between both groups in syncopal attacks. Syncopal attacks were found in 51 patients in younger age group (70.8%) compared to 100 patients (57.5%) in older age group with a *p*-value of 0.052.

HCM as an etiology for SCD was significantly higher in older age group (44 patients with a percentage of 25.3%) compared to only 6 patients (8.3%) in younger age group (*p*-value: 0.003). DCM was also dominant in older age group, as it was the cause of SCD in 42 patients (24.1%) with only 5 patients (6.9%) in younger age group having SCD due to DCM. There was no significant difference between both groups regarding other etiologies of SCD as shown in Table 4.

**Table 4:** Comparing causes of SCD according to age group

Diagnosis	Age group				*p*-value
	Younger age group (*n* = 72)		Older age group (*n *= 147)		
	*N*	%	*N*	%	
HCM	6	8.3	44	25.3	0.0026*
DCM	5	6.9	42	24.1	0.0018*
Congenital long QT Syndrome	4	5.5	24	13.8	0.0626
Congenital CHB	5	6.9	16	9.2	0.5575
Brugada syndrome	5	6.9	11	6.3	0.8622
CPVT	4	5.5	10	5.7	0.9508
Not diagnosed	6	8.3	9	5.2	0.3566
WPW syndrome	4	5.5	11	6.3	0.8114
ARVD	4	5.5	10	5.7	0.9508
Idiopathic VT	3	4.2	6	3.4	0.7608
Primary VF	1	1.4	4	2.3	0.6500
Mitral annular disjunction	0	0	2	1.1	0.3726
Concealed Anteroseptal AP	0	0	3	1.7	0.2667
PVCs from LVOT (Bigeminy)	1	1.4	2	1.1	0.8437
IHD	0	0	2	1.1	0.3726
LV non compaction	0	0	2	1.1	0.3726

*ARVD* Arrhythmogenic right ventricular dysplasia, *CHB* Complete heart block, *CPVT* Catecholamine-mediated ventricular tachycardia, *DCM* Dilated cardiomyopathy, *HCM* Hypertrophic cardiomyopathy, *ICM* Ischemic cardiomyopathy, *IHD* Ischemic heart disease, *LV* Left ventricle, *LVOT* Left ventricular outflow tract, *VF* Ventricular fibrillation, *VT* Ventricular tachycardia, *WPW* Wolff–Parkinson–White

*P < 0.05 is considered significant

### Comparison according to the presence of family history

**Fig. 2:**
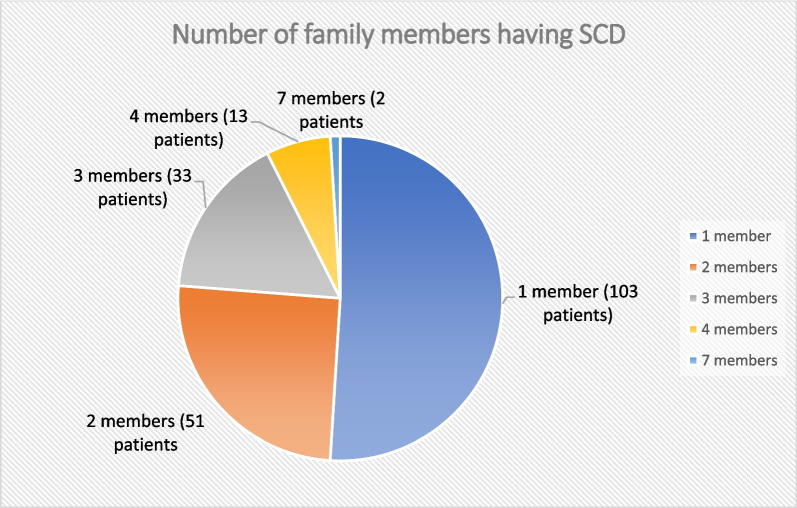
Showing number of family members (for each patient with positive family history) having SCD

Study population was divided into another two groups according to the presence or absence of family history of SCD. Positive family history (FH) group included 202 patients (82.1%) and negative family history (FH) which included 44 patients (17.9%). Number of family members, for each patient, who experienced SCD are shown in Fig. 2.

As shown in Table 5, both groups were similar regarding age groups. Male gender was predominant in negative FH group (41 male patients, 93.2%) in contrast to 103 female patients (51%) versus 99 males (49%) in positive FH group. No significant difference was found in relation to BMI, consanguinity. More patients (52.8%) with positive family history lived in urban areas.

**Table 5:** Comparing demographic data between positive and negative family history groups

Demographic data		Positive FH Group (*n *= 202)	Negative FH Group (*n* = 44)	*p*-value
Age (years)	Mean ± SD	26.01 ± 12.09	26.85 ± 12.31	0.6776
	Range	2–40	4–40	
	2–18 years No. (%)	60 (29.7%)	12 (27.3%)	
	18–40 years No. (%)	142 (70.3%)	32 (72.7%)	
Gender	Males No. (%)	99 (49%)	41 (93.2%)	< 0.0001*
	Females No. (%)	103 (51%)	3 (6.8%)	
BMI (kg/m^2^)	Mean ± SD	22.6 ± 1.41	23.10 ± 1.5	0.5341
	Range	18.5–26.7	19.5–26.7	
Socioeconomic status	High No. (%)	21 (10.4%)	10 (22.7%)	0.6262*
	Moderate No. (%)	152 (75.2%)	18 (40.9%)	< 0.0001*
	Low No. (%)	29 (14.3%)	16 (36.4%)	0.0006*
Residency	Urban areas No. (%)	130 (52.8%)	8 (3.2%)	< 0.0001*
	Rural areas No. (%)	72 (29.2%)	36 (14.6%)	0.0474*
Consanguinity	*N* (%)	84 (41.6) %	16 (36.4%)	0.3810

*BMI* Body mass index

*p < 0.05 is considered significant

It was obvious that HCM, as an etiology of SCD, was more prevalent in positive FH group represented by 46 patients (22.8%) in contrast to only 4 patients (9.1%) having HCM with no family history of SCD (*p*-value: 0.041). On the other side, Brugada syndrome appears to be significantly related to SCD in patients who have no family history of SCD. SCD occurred in 7 Brugada syndrome patients out of 44 of the negative FH group (15.9%) in comparison with 11 Brugada syndrome patients out of 202 of the positive FH group (5.4%) with significant p value of 0.032. There were no other etiologies of SCD showing a significant difference between both groups according to the presence of family history of SCD (Table 6).

**Table 6:** Comparing causes of SCD according to the presence of family history of SCD

Diagnosis	Age group				*p*-value
	Positive FH group (*n* = 202)		Negative FH group (*n* = 44)		
	N	%	*N*	%	
HCM	46	22.8	4	9.1	0.0412*
DCM	36	17.8	11	25	0.2718
Congenital long QT Syndrome	23	11.4	5	11.4	1
Congenital CHB	17	8.4	4	9.1	0.8805
Brugada syndrome	11	5.4	7	15.9	0.0316*
CPVT	13	8.9	1	2.3	0.1380
Not diagnosed	11	6.4	4	9.1	0.5223
WPW syndrome	12	5.9	3	6.8	0.8210
ARVD	12	5.9	2	4.5	0.7160
Idiopathic VT	6	3	1	2.3	0.3865
Primary VF	4	2.9	1	2.3	0.8271
Mitral annular disjunction	2	2.5	0	0	0.2902
Concealed Anteroseptal AP	2	0.9	1	2.3	0.4310
PVCs from LVOT (Bigeminy)	2	0.9	1	2.2	0.4611
IHD	2	0.9	0	0	0.5285
LV non compaction	2	0.9	0	0	0.5285

*ARVD* Arrhythmogenic right ventricular dysplasia, *CHB* Complete heart block, *CPVT* Catecholamine-mediated ventricular tachycardia, *DCM* Dilated cardiomyopathy, *HCM* Hypertrophic cardiomyopathy, *ICM* Ischemic cardiomyopathy, *IHD* Ischemic heart disease, *LV* Left ventricle, *LVOT* Left ventricular outflow tract, *VF* Ventricular fibrillation, *VT* Ventricular tachycardia, *WPW* Wolff–Parkinson–White

*p < 0.05 is considered significant

## Discussion

Prevention of SCD/SCA in the young remains a largely unsolved public health problem. Although the presence of standard cardiovascular risk factors in the young can determine the morbidity and mortality in adulthood, the potential contribution of these risk factors to SCD/SCA in the young has not been fully evaluated. Considerable efforts had been made to understand the background and causes for SCD/SCA, and the differences in its prevalence within and between countries.

In our study, more patients were males constituting 56.9% in the whole studied population, and also, males were predominant in older age group (18–40 years). Similar studies conducted on SCD/SCA cases showed even more male cases. Margey et al. conducted their retrospective registry on 342 SCD/SCA cases in young age and showed that males were 65% [6]. Also, Wisten et al. who conducted their study on 181 cases of SCD/SCA and found that 73% were males and 27% were females [7]. Also, another study by Peterson et al. who studied causes and incidence of SCD/SCA in young population found that the majority were males (83.7%) [8].

Evaluation of circumstances of death revealed that 49.6% of SCD/SCA cases occurred mostly during non-exertion or sleep, while 15.4% cases developed SCD/SCA during practicing exercise. This was near to what was found by Margey et al. study which showed that 45% SCD/SCA cases occurred during non-exertion or sleep and 7.7% occurred during exertion [6]. Also, the prospective study by Quenin et al. who evaluated 103 families with unexplained SCD/SCA before 45 years of age revealed that 40% SCDs occurred at rest and 17% during exercise. The death circumstances remained unknown in 43% SCDs in their study [9].

Investigating the etiology of SCD, hypertrophic cardiomyopathy was the most common cause of SCD/SCA which represented 20.3%. This goes with Peterson et al. study which studied causes and incidence of SCD/SCA in 331 cases young people and found that the most common cause was hypertrophic cardiomyopathy (20.6%) followed by other cardiomyopathies (arrhythmogenic, dilated, noncompaction or restricted), coronary artery anomalies (12%) and unexplained death (9.6%) [8].

Our findings also were supported by the study of Maron et al. which evaluated SCD/SCA in 387 young people less than 35 years. They found that hypertrophic cardiomyopathy was the most common cause of sudden cardiac death (26.4%), followed by dilated cardiomyopathy (20%), arrhythmogenic right ventricular cardiomyopathy (2.8%), long QT syndrome (0.8%), aortic aneurysm (3.1%) and cardiac sarcoidosis (0.8%) [10].

Similarly, the study by Corrado et al. who evaluated 273 cases of SCD/SCA in young population less than 35 years old showed that 72% SCD cases had cardiomyopathies, while 22% of cases had conduction system disorders, ventricular preexcitation and heart block, and in 6% cases there was no evidence of structural diseases. Also, evaluation of causes of SCD/SCA by Margey et al. [6] showed that hypertrophic cardiomyopathy was the common causes of death (14.6%) after sudden arrhythmic death syndrome (26.7%) [11].

This was in contrast to Jayaraman et al. who assessed causes of SCD/SCA in young people, and they found that the most common causes were sudden arrhythmic death syndrome (31%), followed by coronary artery disease (22%), and the least common cause was hypertrophic cardiomyopathy (14%) [12]. Wisten et al. conducted their study on 181 of sudden cardiac death, and they found that the most common diagnosis was coronary artery disease (17.7%) followed by dilated cardiomyopathy (12.2%), hypertrophic cardiomyopathy (10.5%), myocarditis (10.5%) and patients without structural heart disease constituted (21.0%) [7].

The prevalence of hypertrophic cardiomyopathy in our study was significantly higher in the age group between 18 and 40 years (25.3% vs. 8.3%). This was consistent with Berger et al. who investigated the prevalence of hypertrophic cardiomyopathy in the young population, and they found that there were no hypertrophic cardiomyopathy cases in the age group of 0–15 years, in contrast to 30% in the age group between 15 and 45 years [13]. In the retrospective study conducted by Maron et al. to evaluate prevalence with hypertrophic cardiomyopathy, it was more common in the age group between 13 and 30 years, consisting with our results [10]. Our study also confirmed that fact that hypertrophic cardiomyopathy is more common in patients with family history of SCD (22.8% vs. 9.1%). The observation that sudden death can occur in multiple relatives of the same family, and clinical studies in which a family history of HCM-related sudden death emerges as an independent predictor of sudden death, support the principle that family history should be considered a risk factor [13].

In our study, prevalence of dilated cardiomyopathy was 19.1%. It was lower in the age group 2–18 years than in age group between 18 and 40 years [(6.9% vs. 24.9%)]. This was concordant to what was found by Berger et al. who documented the prevalence of idiopathic dilated cardiomyopathy in the young population. They did not have any cases below the age of 15 years, and 67% of their cases were in the age group between 15 and 45 years [14].

Arrhythmogenic right ventricular dysplasia was found in 14 patients (5.7%) in our study, with no difference in prevalence according to age group. On the contrary, Holst et al. studied causes of SCD in Denmark and found that ARVD was the most common causes of SCD between ages 12–35 years [15]. Also, Quarta et al., who assessed 50 patients with AVRD, found that ARVD was more common in younger patients between 14 and 20 years of age [16].

Long QT syndrome as an important cause of SCD was only represented with 11.4% in our study, with no difference in prevalence according to age group or the presence of family history. On the other hand, a retrospective study by Skinner et al., who evaluated causes SCD/SCA in the young (1–40 years), revealed that 66% of the cases was due to long QT syndrome. In addition, they had 4 cases of SCD due to dilated cardiomyopathy, 4 cases due to poisoning, 4 cases due to myocarditis, and 9 cases of SCD due to unexplained sudden cardiac death. [17].

The prevalence of Brugada syndrome in our study was 6.8%. It was also found in our study that fewer cases of SCD had a positive family history of SCD. This was of course inconsistent with the results of the large meta-analysis done by Rattanawong et al. which included 22 studies examining the relation between Brugada syndrome and occurrence of major arrhythmic events, and they concluded that the presence of a history of SCD among family members of age younger than 40 years was associated with a higher risk of major arrhythmic events [18]. The results of the current study can be explained by the relatively small number of patients diagnosed by Brugada syndrome in the current study.

## Limitations

Our sample size was relatively small; however, our cases were recruited from 5000 records for tertiary arrhythmia clinic and they represented wide spectrum of population characteristics in Egypt. Other limitation is unavailability of genetic screening of family members and autopsy; however, this was overcome by other integrated noninvasive investigations.

## Conclusions

Family history of SCD was the most common risk factor of SCD, and accordingly, it may be a reliable predictor of high risk for SCD/SCA cases in the young. The most common cause of SCD was hypertrophic cardiomyopathy, followed by dilated cardiomyopathy. This was true for the age group between 18 and 40 years. Their prevalence was significantly higher than that of the age group between 2 and 18 years. Hypertrophic cardiomyopathy was found to be more common in patients with positive family history of SCD/SCA.

## Data Availability

The datasets used and/or analyzed during the current study are available from the corresponding author on reasonable request.

## Abbreviations

ARVD: Arrhythmogenic right ventricular dysplasia
BUN: Blood urea nitrogen
CBC: Complete blood count
CHB: Complete heart block
CPVT: Catecholamine-mediated polymorphic ventricular tachycardia
CRT-D/P: Cardiac resynchronization therapy defibrillator/pacemaker
CT: Computed tomography
DCM: Dilated cardiomyopathy
DDD: Dual camber pacemaker
ECG: Electrocardiogram
FH: Family history
HCM: Hypertrophic cardiomyopathy
ICD: Implantable cardioverter defibrillator
ICM: Ischemic cardiomyopathy
IHD: Ischemic heart disease
LVOT: Left ventricular outflow tract
MRI: Magnetic resonance imaging
PVCs: Premature ventricular contraction
RVOT: Right ventricular outflow tract
VF: Ventricular fibrillation
VT: Ventricular tachycardia
WPW: Wolff–Parkinson–White
